# Micafungin prophylaxis for acute leukemia patients undergoing induction chemotherapy

**DOI:** 10.1186/s12885-019-5557-9

**Published:** 2019-04-16

**Authors:** Hyunkyung Park, Jeonghwan Youk, Dong-Yeop Shin, Junshik Hong, Inho Kim, Nam Joong Kim, Jeong-Ok Lee, Soo-Mee Bang, Sung-Soo Yoon, Wan Beom Park, Youngil Koh

**Affiliations:** 1 Department of Internal Medicine, Seoul National University Hospital, 101 Daehak-ro, Jongno-gu, Seoul, 03080 South Korea; 2 Korea Advanced Institute of Science and Technology, Daejeon, South Korea; 3 Cancer Research Institute, Seoul National University College of Medicine, Seoul, South Korea; 4 Biomedical Research Institute, Seoul National University Hospital, Seoul, South Korea; 5 Department of Internal Medicine, Seoul National University Bundang Hospital, Seongnam, South Korea

**Keywords:** Acute leukemia, Prophylaxis, Antifungal agent, Micafungin, Posaconazole

## Abstract

**Background:**

Micafungin is a well-tolerated and effective prophylactic antifungal agent used in hematologic diseases. In this prospective trial, we evaluated the efficacy and safety of prophylactic micafungin during first induction chemotherapy in patients with acute leukemia. We also compared outcomes of prophylactic micafungin with those of prophylactic posaconazole in acute myeloid leukemia (AML).

**Methods:**

Medically fit patients with newly diagnosed acute leukemia received 50 mg micafungin intravenously once daily from the initiation of first induction chemotherapy to recovery of neutrophil count, suspected fungal infection, or unacceptable drug-related toxicity (Clinicaltrials.gov number, NCT02440178). The primary end point was incidence of invasive fungal infection, and the secondary end points were adverse events of prophylactic micafungin and mortality during induction therapy.

**Results:**

The 65 patients (median age = 51 years, male:female = 34:31) enrolled in this study had diagnoses of AML (33, 50.8%), acute lymphoblastic leukemia (31, 47.7%), and acute biphenotypic leukemia (1, 1.5%). Median duration of micafungin treatment was 24 days (range 1–68), with proven invasive fungal disease in one patient (1.5%) and possible fungal infection in two patients (3.1%). Three of the patients (4.6%) experienced the following adverse events, but all events were tolerable: liver function abnormality (Grade 2, *n* = 1; Grade 3, n = 1) and allergic reaction (Grade 2, n = 1). Three patients died during induction therapy, and invasive aspergillosis pneumonia was the cause of death for one of those patients. Overall, 19 patients (29.2%) discontinued prophylactic micafungin, and 18 (27.7%) patients switched to another antifungal agent. We observed no fungal infections caused by amphotericin B-resistant organisms. In AML patients, outcomes of prophylactic micafungin during induction chemotherapy did not differ significantly with those of prophylactic posaconazole with regard to incidence of fungal infections, rate of discontinuation, or safety.

**Conclusions:**

Our study demonstrates that prophylactic micafungin is safe and effective in patients with acute leukemia undergoing induction chemotherapy. Outcomes in patients with AML were similar to those of prophylactic posaconazole, indicating the usefulness of micafungin as a prophylactic antifungal agent during induction chemotherapy for AML.

**Trial registration:**

Clinicaltrials.gov NCT02440178, registered May 12th 2015.

## Background

Over the past decades, treatment-related mortality (TRM) rates during induction chemotherapy in patients with acute leukemia have decreased because of improved supportive care [1]. However, according to reports by the SWOG cancer research network and MD Anderson Cancer Center, TRM within 28 days after initiation of induction chemotherapy remains at approximately 3–4% [2]. Infection is the major cause of TRM and patient transfer to the intensive care unit during this period [3]. In particular, invasive fungal infections are common causes of morbidity and mortality in patients with acute leukemia. The estimated incidence of proven/probable invasive fungal disease after diagnosis is 11.1% at 100 days [4, 5]. A prospective, observational study of patients with acute myeloid leukemia (AML) reported that the incidence rate of fungal infection during induction therapy was approximately 34.6%, and presence of an invasive fungal infection during induction independently predicted worse survival [6]. Strategies to address this problem include diagnostic laboratory tests, computed tomography scan for early detection, and early treatment [7].

Earlier work in this area has demonstrated the usefulness of prophylactic antifungal agents in patients with acute leukemia, with the choice of antifungal agent used based on type of leukemia, patient characteristics, and type of treatment [8]. Most patients with acute leukemia undergoing induction chemotherapy or hematopoietic stem cell transplantation (HSCT) are classified as high risk, with aspergillosis species as one of the key pathogens [8]. Prophylactic fluconazole decreases the rate of fungal infections during induction chemotherapy compared to placebo [9] but has no activity against aspergillosis species and certain strains of *Candida*, including *C. krusei* and *C. glabrata* [10]. More recently, the use of anti-mold agents, such as posaconazole and echinocandins has been shown to decrease mortality in patients with acute leukemia at high risk for invasive fungal infections. The European Conference on Infections in Leukemia guidelines recommend posaconazole prophylaxis during induction therapy in acute leukemia [4, 11]; however, the use of posaconazole is limited because its absorption is influenced by genetic polymorphisms, gastrointestinal pH, and diet [12–15].

Micafungin, which is the only echinocandin used as a prophylactic antifungal agent in hematological diseases, has demonstrated good efficacy and tolerable safety in patients undergoing HSCT [16–19]. However, data are limited on the prophylactic use of micafungin during induction chemotherapy in acute leukemia [20–22].

Therefore, this prospective trial evaluated the efficacy and safety of micafungin as a prophylactic antifungal agent for patients with acute leukemia during induction chemotherapy. Additionally, we compared these efficacy and safety outcomes with those of prophylactic posaconazole, which was previously evaluated in an observational study of patients with AML in the same institution [23].

## Methods

### Study design

In this prospective, single-arm, open-label study (Clinicaltrials.gov number, NCT02440178), we enrolled patients with newly diagnosed acute leukemia (AML, acute lymphoblastic leukemia [ALL], and acute biphenotypic leukemia [ABL]) who received intensive induction chemotherapy at Seoul National University Hospital (SNUH) and Seoul National University Bundang Hospital from September 2015 through June 2017. All patients enrolled in the study provided written informed consent. Inclusion criteria were as follows: 1) ≥ 18 years old; 2) Acute leukemia diagnosed by bone marrow examination; 3) intensive induction chemotherapy; 4) Eastern Cooperative Oncology Group performance status score ≤ 2; and 5) serum creatinine and bilirubin levels < 1.5 times the upper limit of the reference range for our laboratory. Exclusion criteria were as follows: 1) suspected fungal infection 30 days before initiation of induction chemotherapy; 2) history of hypersensitivity to echinocandin; 3) diagnosis of other malignancy in the previous 5 years; 4) previous chemotherapy, radiation, or immunosuppressive treatment; 5) immunodeficiency disease; 6) pregnant or breastfeeding; 7) uncontrolled seizures or mental illness; 8) acute myocardial infarction, uncontrolled arrhythmia, or low ejection fractions (< 40%); 9) previous organ transplantation; and 10) interstitial lung disease.

**Fig. 1:**
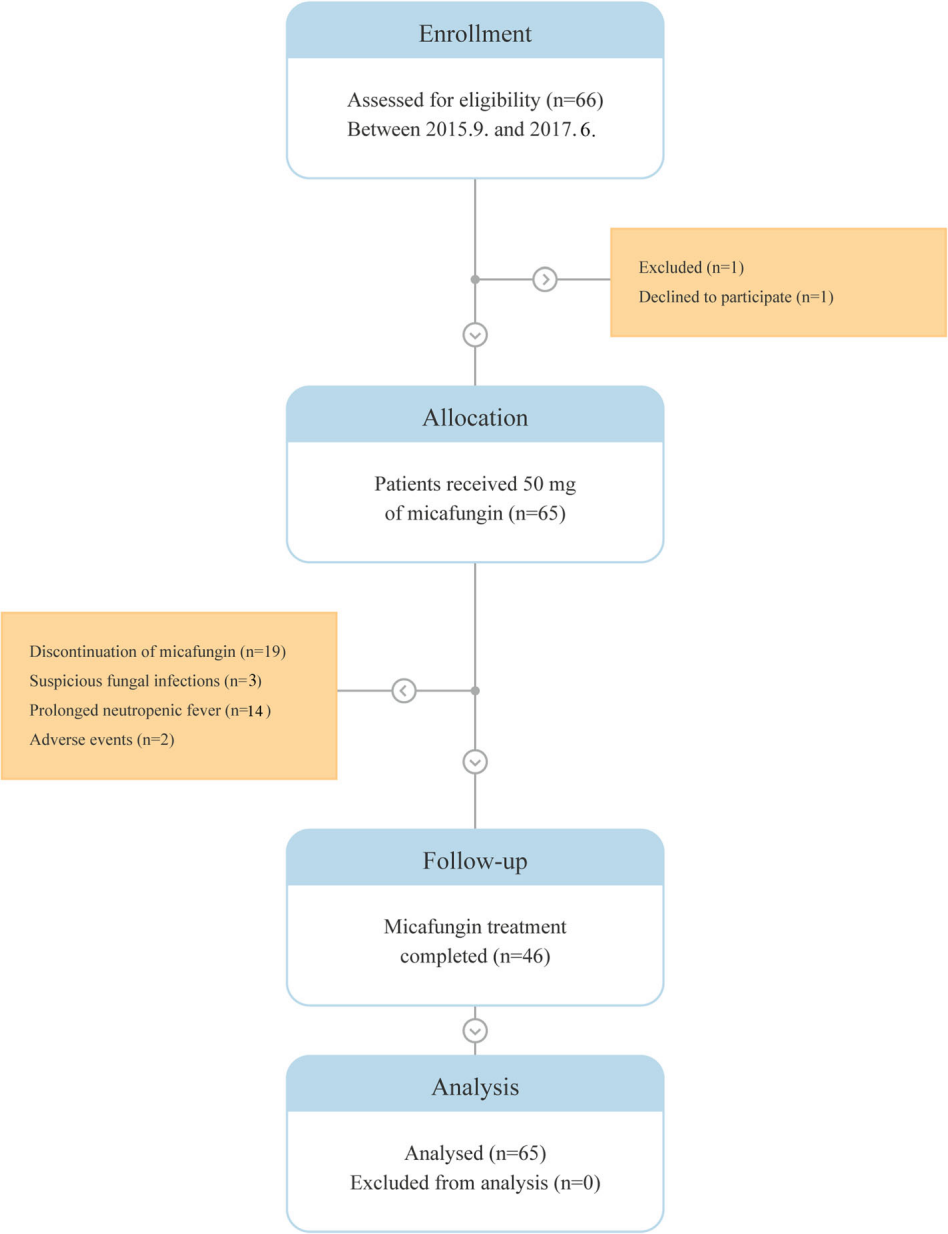
The consort diagram of study

Patients received 50 mg micafungin intravenously once daily from the initiation of induction chemotherapy to recovery of neutrophil count (absolute neutrophil count > 500/μg for three consecutive days), suspected fungal infection, or occurrence of drug-related toxicity. The primary end point was incidence of invasive fungal infection, and the secondary end points were adverse events of prophylactic micafungin and mortality during induction chemotherapy. The study flow diagram is shown in Fig. 1. Patients were followed up for 6 and 12 weeks after the initiation of induction chemotherapy for the occurrence of fungal infection and survival, respectively.

### The determination of sample size

The previous study showed that the incidence of fungal infections was about 29.2% in prophylactic fluconazole group and 13.8% in posaconazole group [24]. We hypothesized that the incidence of fungal infections in the prophylactic micafungin group was reduced by 15.4% (assuming that the incidence is similar to that of the prophylactic posaconazole group) from that of prophylactic fluconazole group. Therefore, a sample size of 65 patients was required for this study based on a statistical power of 80% and a significance level of 5%, including a dropout rate of 10%.

### Diagnosis of invasive fungal infection

For all patients, baseline chest x-rays were obtained within 7 days after the initiation of induction chemotherapy. *Aspergillus* infection was diagnosed according to the criteria for invasive pulmonary aspergillosis of the European Organization for Research and Treatment of Cancer/Invasive Fungal Infections Cooperative Group and the National Institute of Allergy and Infectious Diseases Mycoses Study Group (EORTC/MSG) Consensus Group [25]. An invasive fungal infection was defined as “proven” by a positive culture for fungus with symptoms and signs of a fungal infection, as “probable” by direct or indirect detection (galactomannan antigen or serum *β*–D-glucan) with clinical and radiographic findings, or as “possible” if sufficient clinical evidence for fungal infection was present without mycological evidence.

### Comparison of outcomes of prophylactic micafungin with those of previously performed prophylactic posaconazole trial

We compared the efficacy and safety of prophylactic micafungin with those of prophylactic posaconazole during induction chemotherapy in AML patients treated at SNUH [23]. This prospective observational trial compared plasma posaconazole concentrations in patients who received posaconazole as an oral suspension versus tablet. From September 2014 to April 2017, we enrolled patients who received prophylactic posaconazole while undergoing induction chemotherapy for AML or myelodysplastic syndrome. Among these patients, we selected patients with AML who met the inclusion criteria of our study for comparison with patients who received prophylactic micafungin in the present study.

### Statistical analysis

Categorical variables were compared using Pearson’s chi-square test or Fisher’s exact test, as appropriate. Cumulative incidence of fungal infection and the time from initiation of micafungin treatment to a switch to another antifungal agent were evaluated by Kaplan–Meier analysis. All analyses were performed using SPSS for Windows, version 23.0 (IBM; Armonk, NY, USA); All statistical tests were two-sided, and significance was defined as *P* < 0.05.

### Ethical considerations

This study was approved by the institutional review board at Seoul National University Hospital (IRB; H-1412-022-631) and was conducted in accordance with the guidelines of the Declaration of Helsinki for biomedical research. Informed consent was obtained from all participants.

## Results

### Patient characteristics

Sixty-five patients were enrolled in this study (Table 1). Median patient age was 51 years (range, 18–84 years). Diagnoses were AML (*n* = 33, 50.8%), ALL (31, 47.7%), and ABL (*n* = 1, 1.5%). All patients received intensive induction chemotherapy.

**Table 1:** Baseline patient characteristics

Patient characteristics	Patients (*n* = 65)
Median age, years (range)	51 (18–84)
Age group, *n* (%)	
< 60 years	48 (73.8)
≥ 60 years	17 (26.2)
Sex, *n* (%)	
Male	34 (52.3)
Female	31 (47.7)
Disease, *n* (%)	
AML	33 (50.8)
De novo AML	23 (35.4)
Secondary AML^a^	10 (15.4)
ALL	31 (47.7)
Philadelphia-positive	3 (4.6)
Philadelphia-negative	28 (43.1)
ABL	1 (1.5)
Risk group, *n* (%) [1, 36]	
Favorable	3 (4.6)
Intermediate	29 (44.6)
Poor	30 (46.2)
Unknown	3 (4.6)
Risk group (AML only), *n* (%) [1]	
Favorable	3 (9.1)
Intermediate	16 (48.5)
Poor	12 (36.4)
Unknown	2 (6.0)
Induction chemotherapy, n (%)	
AID-based regimen	30 (46.2)
Modified FLAI	3 (4.6)
VPD-based regimen	30 (46.2)
ADVP-based regimen	2 (3.1)
Median BM blast percentage at diagnosis (range)	80.0 (12.9–98.5)
Median duration of micafungin treatment, days (range)	24 (1–68)
Median time from induction chemotherapy to severe neutropenia (ANC < 500/μL), days (range)	24 (11–84)

Abbreviations: *AML* Acute myeloid leukemia, *ALL* Acute lymphoblastic leukemia, *ABL* Acute biphenotypic leukemia, *AID* Cytarabine + idarubicin, *FLAI* Fludarabine + cytarabine + idarubicin, *VPD* Vincristine + prednisolone + daunorubicin, *ADVP* Cytarabine + daunorubicin + vincristine + prednisolone, *BM* Bone marrow, *ANC* Absolute neutrophil count; ^a^Secondary AML (10) included AML transformation from myelodysplastic syndrome (8), AML transformation from chronic myelomonocytic leukemia (1) and treatment-related AML (1)

### Efficacy and adverse events of micafungin prophylaxis

**Fig. 2:**
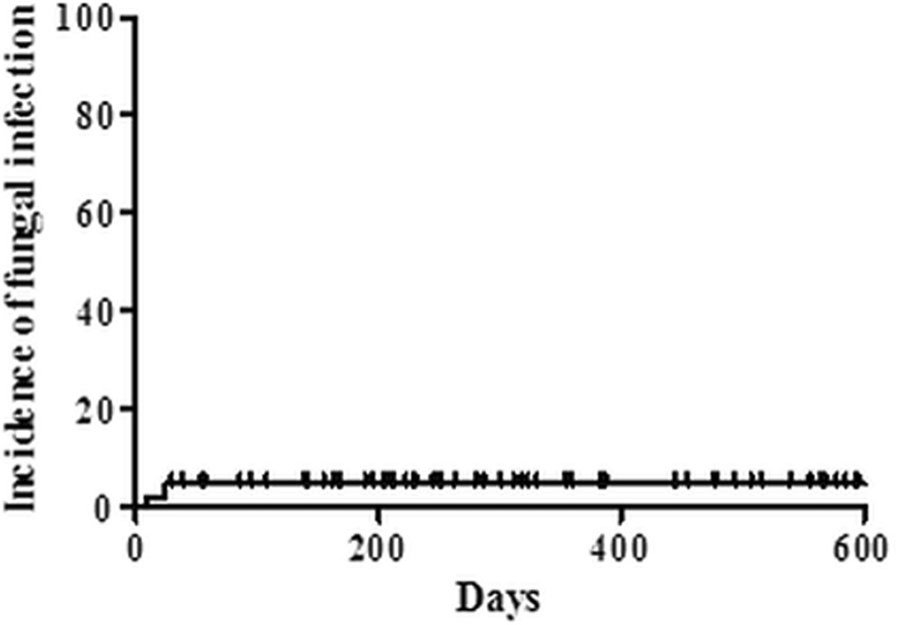
Kaplan–Meier curve of cumulative incidence of fungal infection during induction chemotherapy

The median duration of micafungin treatment was 24 days (range 1–68 days) (Table 1). During induction chemotherapy, invasive fungal infection was detected in three of the 65 patients (4.6%), with proven invasive fungal disease in one patient (1.5%) and possible fungal infection in two patients (3.1%) (Table 2 and Fig. 2). The patient with proven invasive fungal disease had an initial diagnosis of poor-risk AML (transformation of myelodysplastic syndrome to AML) and received cytarabine and idarubicin induction chemotherapy. During treatment, the patient developed uncontrolled fever, and invasive pulmonary aspergillosis was diagnosed by bronchoscopic biopsy on day 25 after initiation of induction chemotherapy. Although she was treated with voriconazole, the fungal pneumonia gradually worsened, and diffuse alveolar hemorrhage developed. This patient died in intensive care unit due to pneumonia septic shock and aggravated diffuse alveolar hemorrhage. One patient with possible fungal infection had a diagnosis of poor-risk AML and was treated with cytarabine and idarubicin induction chemotherapy. She experienced erythematous nodular skin lesions and pneumonia, and fungal infection was suspected on day 23 after initiation of chemotherapy. After treatment with liposomal amphotericin B, the skin lesions and pneumonia improved. The other patient with possible fungal infection had a diagnosis of Philadelphia-positive ALL and received vincristine, prednisolone, daunorubicin, L-asparaginase, and imatinib (VPDL + imatinib) chemotherapy. Fungal pneumonia was suspected based on computed tomography imaging on day 9 after initiation of chemotherapy. The pneumonia was treated with voriconazole and gradually improved.

**Table 2:** Discontinuation and adverse events of prophylactic micafungin during first induction chemotherapy

	Number of patients (%)
Incidence of fungal infection	3/65 (4.6)
Proven fungal infection	1/65 (1.5)
Probable fungal infection	0
Possible fungal infection	2/65 (3.1)
Discontinuation of prophylactic micafungin	19/65 (29.2)
Cause of discontinuation	
Suspected fungal infections	3/65 (4.6)
Prolonged neutropenic fever	14/65 (21.5)
Adverse events	2/65 (3.1)
Micafungin treatment completed	46/65 (70.8)
Switch to another antifungal agent	18/65 (27.7)
Adverse events related to micafungin	3/65 (4.6)
Liver function test abnormality	2/65 (3.1)
Grade 2	1/65 (1.5)
Grade 3	1/65 (1.5)
Allergic reaction	1/65 (1.5)
Grade 2	1/65 (1.5)
No adverse event	62/65 (95.4)
Cause of mortality during induction chemotherapy	3/65 (4.6)
Fungal infection (proven)	1/65 (1.5)
Respiratory failure (pneumonia and pulmonary edema)	1/65 (1.5)
Septic shock	1/65 (1.5)

**Fig. 3:**
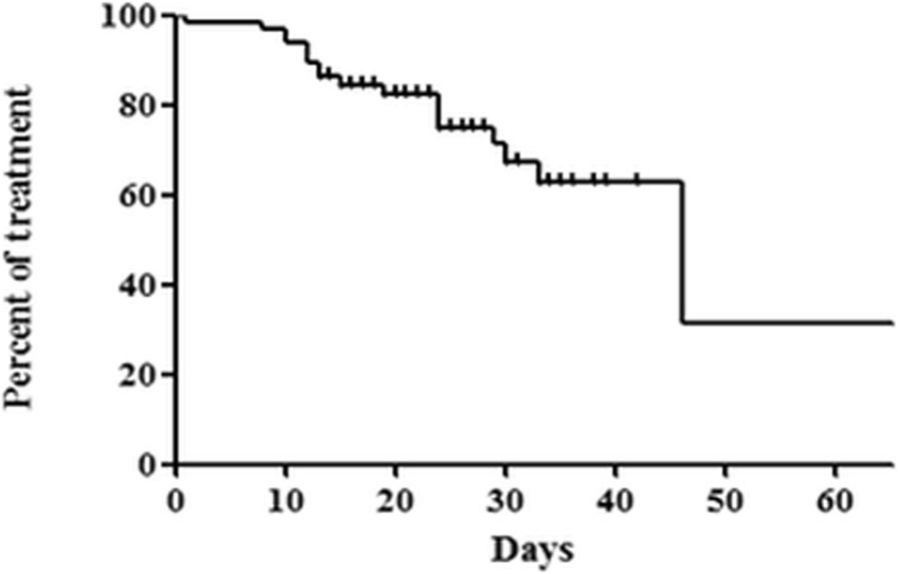
Kaplan–Meier curve showing time from initiation of prophylactic micafungin to switch to another antifungal agent

Nineteen of the 65 patients (29.2%) discontinued prophylactic micafungin because of prolonged neutropenic fever (*n* = 14, 21.5%), fungal infection (*n* = 3, 4.6%), or adverse events (*n* = 2, 3.1%) (Table 2). Of these 19 patients, 18 patients changed to other antifungal agents; 1 patient changed to itraconazole; 1 patient changed to posaconazole, 2 patients changed to voriconazole, and 14 patients changed to liposomal amphotericin B. The reasons for the change to other antifungal agents were as follows: 3 patients were suspected of having fungal infections; 14 patients experienced prolonged neutropenic fever, and 1 patient developed a grade 2 allergic reaction. The time from initiation of micafungin to a switch to another antifungal agent was 46 days (95% confidence interval [CI], 27–64 days) (Fig. 3).

Three of the 65 patients (4.6%) experienced the following micafungin-related adverse events: Grade 2 or 3 liver function abnormality (n = 2, 3.1%) and Grade 2 allergic reaction (*n* = 1). Micafungin treatment was interrupted for the patient with Grade 3 liver function abnormality, and liver enzyme levels gradually improved, returning to the reference range after 22 days. The patient with Grade 2 liver function abnormality continued on prophylactic micafungin, and liver enzyme levels returned to the reference range. The patient with the allergic reaction (sudden chest discomfort, dizziness, abdominal pain, and drop in systolic blood pressure from 140 mmHg to 93 mmHg) recovered after discontinuation of micafungin and treatment with an antihistamine.

In three patients, chemotherapy-related death occurred due to invasive aspergillosis pneumonia (*n* = 1, on day 39), septic shock (n = 1, on day 45), or respiratory failure caused by pulmonary edema and pneumonia (*n* = 1, on day 30). No patients experienced fungal infections caused by amphotericin B-resistant organisms after induction chemotherapy.

### Comparison of micafungin prophylaxis with posaconazole prophylaxis in patients with newly diagnosed AML receiving induction chemotherapy

**Fig. 4:**
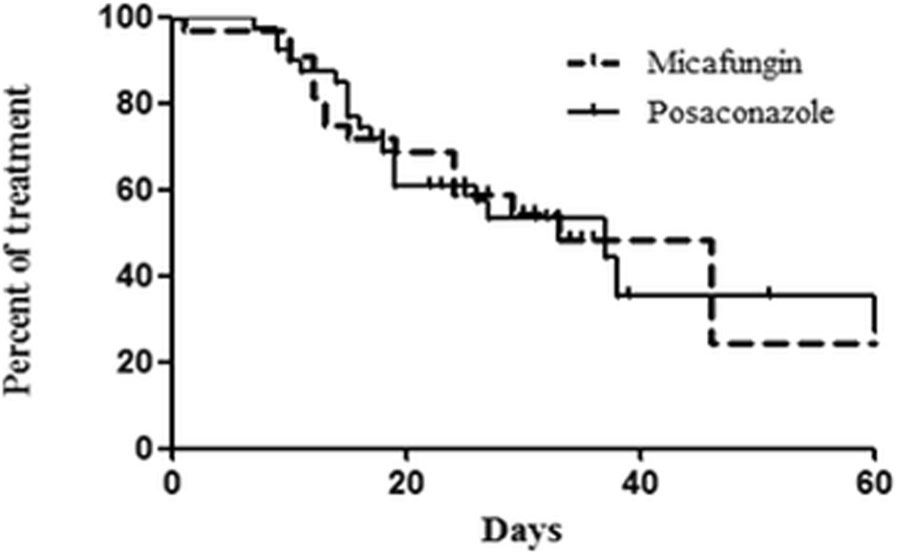
Kaplan–Meier curve showing time from initiation of prophylactic micafungin or posaconazole to a switch to another antifungal agent

A subgroup analysis of AML patients in our study (*n* = 32) showed that two patients (6.3%) experienced invasive fungal infections (proven infection, n = 1; possible infection, *n* = 1). Seventeen of the patients (53.1%) discontinued prophylactic antifungal treatment, and 16 (50.0%) switched to another antifungal agent. A comparison with AML patients who received posaconazole at SNUH in a previous study [23] showed no significant differences in baseline characteristics except for bone marrow blast percentage at diagnosis (*P* = 0.02) (Table 3). No significant differences between the two groups were found regarding incidence of total invasive fungal infections (1 proven and one possible fungal infection in the micafungin group vs. 2 probable fungal infections in the posaconazole group; *P* = 1.000); discontinuation of the prophylactic agent (17/32 [53.1%] for the micafungin group vs. 20/39 [51.3%] for the posaconazole group; *P* = 0.877); or switch to another antifungal agent (16/32 [50.0%] for the micafungin group vs. 20/39 [51.3%] for the posaconazole group; *P* = 0.914) (Table 4). Additionally, the incidence of adverse events did not differ significantly between groups (2/32 patients [6.3%] in the micafungin group vs. 4/39 patients [10.3%] in the posaconazole group; *P* = 0.683). All adverse events in the posaconazole group were liver function abnormalities. Finally, no significant differences between the two groups were found regarding mortality during induction chemotherapy (3/32 patients [9.4%] in the micafungin group vs. 2/39 patients [5.1%] in the posaconazole group; *P* = 0.652) or time from the initiation of micafungin to switch to another antifungal agent (33 days for the micafungin group vs. 37 days for the posaconazole group; *P* = 0.900) (Fig. 4).

**Table 3:** Clinicopathologic characteristics of patients with acute myeloid leukemia treated with prophylactic micafungin or posaconazole at SNUH

Patient characteristics	Micafungin (n = 32)	Posaconazole (*n* = 39)	P -value
Median age, years (range)	57 (21–84)	52 (18–73)	0.408
Sex, n (%)			
Male	18 (56.3)	19 (48.7)	
Female	14 (43.8)	20 (51.3)	0.527
Disease, *n* (%)			
De novo AML	23 (71.9)	33 (84.6)	0.191
Secondary AML	9 (28.1)	6 (15.4)	
Induction chemotherapy, *n* (%)			
AID-based regimen	29 (90.6)	32 (82.1)	0.495
Modified FLAI	3 (9.4)	7 (17.9)	
Median BM blast percentage at diagnosis (range)	62.6 (12.9–95.4)	32.2 (17.7–99.0)	0.020
Median duration of micafungin treatment, days (range)	26.0 (1.0–68.0)	25.0 (7.0–95.0)	0.782
Median time from chemotherapy to severe neutropenia, days (range)	28.5 (11.0–84.0)	28.0 (7.0–139.0)	0.776

Abbreviations: *AML* Acute myeloid leukemia, *AID* Cytarabine + idarubicin, *FLAI* Fludarabine + cytarabine + idarubicin, *BM* Bone marrow

**Table 4:** Outcomes of prophylactic micafungin vs. posaconazole during first induction chemotherapy in newly diagnosed AML patients

	Micafungin, *n* (%)	Posaconazole, *n* (%)	*P*-value
Incidence of fungal infection	2/32 (6.3)	2/39 (5.1)	1.000
Proven fungal infection	1/32 (3.1)	0	
Probable fungal infection	0	2/39 (5.1)	
Possible fungal infection	1/32 (3.1)	0	
Discontinuation of prophylactic antifungal agent	17/32 (53.1)	20/39 (51.3)	0.877
Cause of discontinuation			
Suspected fungal infection	2/32 (6.3)	2/39 (5.1)	
Prolonged neutropenic fever	13/32 (40.6)	17/39 (43.6)	
Any adverse event	2/32 (6.3)	0	
Oral mucositis	0	1/39 (2.6)	
Antifungal treatment completed	15/32 (46.9)	19/39 (48.7)	
Change to another antifungal agent	16/32 (50.0)	20/39 (51.3)	0.914
Adverse event of prophylactic antifungal agent	2/32 (6.3)	4/39 (10.3)	0.683
Liver function test abnormality	1/32 (3.1)	4/39 (10.3)	
Allergic reaction (Grade 2)	1/32 (3.1)	0	
No adverse event	30/32 (93.8)	35/39 (89.7)	
Cause of mortality during chemotherapy	3/32 (9.4)	2/39 (5.1)	0.652
Fungal infection (proven)	1/32 (3.1)	0	
Respiratory failure (pneumonia, pulmonary edema)	1/32 (3.1)	1/39 (2.6)	
Septic shock	1/32 (3.1)	1/39 (2.6)

## Discussion

Despite improvement in supportive care, invasive fungal infections remain among the most common causes of mortality in patients with high risk hematologic diseases; therefore, prophylactic antifungal treatment is used in these patients [8]. In acute leukemia, induction chemotherapy is a period in which patients are particularly vulnerable to fungal infections [8]. Considering drug toxicity, coverage of pathogens, and safety, current guidelines strongly recommend the use of prophylactic posaconazole during induction chemotherapy (Grade 1A recommendation) [11].

Previous clinical trials have demonstrated the efficacy and safety of prophylactic micafungin for hematologic malignancies. For example, Van Burik, et al. reported that the overall efficacy of micafungin was superior to that of fluconazole after HSCT (treatment success rate: 80% for micafungin vs. 73.5% for fluconazole; *P* = 0.03) [19]. A previous Korean study reported superior cost-effective outcomes with prophylactic micafungin compared with prophylactic fluconazole during HSCT (total cost difference: 95,511,000 Korean Won per 100 patients) [26]. In addition, a multicenter, randomized, phase III trial also found that micafungin was as effective as itraconazole in the prevention of invasive fungal infections in patients undergoing HSCT (treatment success rate: 92.6% for micafungin vs. 94.6% for itraconazole; *P* = 0.48) and was also better tolerated (drug-related adverse events: 11% for micafungin vs. 39% for itraconazole; *P* < 0.001) [27]. Finally, a meta-analysis of randomized controlled trials showed significantly higher success rates for micafungin compared with triazole (fluconazole, voriconazole and itraconazole) in patients undergoing intensive chemotherapy or HSCT (relative risk [RR] = 1.15) and fewer severe adverse events (RR = 0.45; *P* = 0.0105) [28].

The results of our study indicate that micafungin may be a useful prophylactic antifungal agent in patients with acute leukemia during induction chemotherapy. In patients with AML, prophylactic micafungin was not inferior to posaconazole with regard to efficacy or safety. In addition, the adverse events of micafungin were tolerable.

Moreover, micafungin has several advantages as a prophylactic antifungal agent. Because it is administered intravenously, micafungin is useful for patients undergoing intensive chemotherapy, which causes side effects that interfere with oral intake (e.g., severe oral mucositis or nausea/vomiting). Micafungin also has a low rate of drug-drug interactions compared with posaconazole, which inhibits cytochrome P450 enzymes [15, 29] and could therefore interact with drugs such as histamine H2 receptor antagonists, proton pump inhibitors, and calcium channel blockers. Especially, micafungin could overcome the severe neurotoxicity which could increase through interaction between antifungal azole and vincristine (one of the important drug in ALL) [30]. Furthermore, plasma levels of posaconazole must be monitored to determine bioavailability, which is affected by diet, mucositis, genetic polymorphisms, gastrointestinal pH, as well as other drugs [12–15]. In contrast, micafungin has a wide safety range, and plasma levels of micafungin do not correlate with degree of hepatic or renal dysfunction [31].

Micafungin use is limited by its lack of activity against certain species of fungus including *Cryptococcus*, *Coccidioides*, Zygomycetes, and *Scedosporium* species [32]. Although the rates of fusariosis and zygomycosis infections have recently increased because of the extensive use of antifungal prophylaxis, most fungal infections in acute leukemia patients are still caused by *Aspergillus* species (55–58%) and *Candida* species (26–40%) [32–34], which can be successfully treated with micafungin. In addition, safety is also an important concern when selecting a prophylactic antifungal agent. For example, a prospective, randomized study comparing fluconazole with the broad-spectrum antifungal agent amphotericin B for antifungal prophylaxis found an increased risk of nephrotoxicity amphotericin B but no increase in efficacy [35]. Therefore, not only the efficacy but also the safety should be considered when selecting a prophylactic antifungal agent.

Limitations of our study include its small sample size and the single-arm study design. The observational study of posaconazole used for comparison also had a small sample size; therefore, large-scale studies are needed to confirm our results. Nevertheless, we believe this study could be helpful for the selection of antifungal agents in patient with acute leukemia treated with first-line induction chemotherapy.

## Conclusions

In conclusion, our study demonstrates that prophylactic micafungin is safe and effective in patients with acute leukemia undergoing induction chemotherapy. In patients with AML, the effectiveness and tolerability of micafungin is similar to those of posaconazole.

## Abbreviations

ABL: Acute biphenotypic leukemia
ALL: Acute lymphoblastic leukemia
AML: Acute myeloid leukemia
CI: Confidence interval
HSCT: Hematopoietic stem cell transplantation
RR: Relative risk
SNUH: Seoul National University Hospital
TRM: Treatment-related mortality

## References

[1] Dohner H, Estey E, Grimwade D, Amadori S, Appelbaum FR, Buchner T, Dombret H, Ebert BL, Fenaux P, Larson RA (2017). Diagnosis and management of AML in adults: 2017 ELN recommendations from an international expert panel. Blood.

[2] Othus M, Kantarjian H, Petersdorf S, Ravandi F, Godwin J, Cortes J, Pierce S, Erba H, Faderl S, Appelbaum FR (2014). Declining rates of treatment-related mortality in patients with newly diagnosed AML given 'intense' induction regimens: a report from SWOG and MD Anderson. Leukemia.

[3] Buckley SA, Othus M, Estey EH, Walter RB (2015). The treatment-related mortality score is associated with non-fatal adverse events following intensive AML induction chemotherapy. Blood cancer journal.

[4] Cornely OA, Maertens J, Winston DJ, Perfect J, Ullmann AJ, Walsh TJ, Helfgott D, Holowiecki J, Stockelberg D, Goh YT (2007). Posaconazole vs. fluconazole or itraconazole prophylaxis in patients with neutropenia. N Engl J Med.

[5] Hammond SP, Marty FM, Bryar JM, DeAngelo DJ, Baden LR (2010). Invasive fungal disease in patients treated for newly diagnosed acute leukemia. Am J Hematol.

[6] Tang JL, Kung HC, Lei WC, Yao M, Wu UI, Hsu SC, Lin CT, Li CC, Wu SJ, Hou HA (2015). High incidences of invasive fungal infections in acute myeloid leukemia patients receiving induction chemotherapy without systemic antifungal prophylaxis: a prospective observational study in Taiwan. PLoS One.

[7] Segal BH, Almyroudis NG, Battiwalla M, Herbrecht R, Perfect JR, Walsh TJ, Wingard JR (2007). Prevention and early treatment of invasive fungal infection in patients with cancer and neutropenia and in stem cell transplant recipients in the era of newer broad-spectrum antifungal agents and diagnostic adjuncts. Clinical infectious diseases : an official publication of the Infectious Diseases Society of America.

[8] Pagano L, Busca A, Candoni A, Cattaneo C, Cesaro S, Fanci R, Nadali G, Potenza L, Russo D, Tumbarello M (2017). Risk stratification for invasive fungal infections in patients with hematological malignancies: SEIFEM recommendations. Blood Rev.

[9] Rotstein C, Bow EJ, Laverdiere M, Ioannou S, Carr D, Moghaddam N (1999). Randomized placebo-controlled trial of fluconazole prophylaxis for neutropenic cancer patients: benefit based on purpose and intensity of cytotoxic therapy. The Canadian fluconazole prophylaxis study group. Clinical infectious diseases : an official publication of the Infectious Diseases Society of America.

[10] Vazquez L (2016). Antifungal prophylaxis in immunocompromised patients. Mediterranean journal of hematology and infectious diseases.

[11] Maertens J, Marchetti O, Herbrecht R, Cornely OA, Fluckiger U, Frere P, Gachot B, Heinz WJ, Lass-Florl C, Ribaud P (2011). European guidelines for antifungal management in leukemia and hematopoietic stem cell transplant recipients: summary of the ECIL 3--2009 update. Bone Marrow Transplant.

[12] Suh HJ, Yoon SH, Yu KS, Cho JY, Park SI, Lee E, Lee JO, Koh Y, Song KH, Choe PG, et al. The genetic polymorphism UGT1A4*3 is associated with low Posaconazole plasma concentrations in hematological malignancy patients receiving the Oral suspension. Antimicrob Agents Chemother. 2018. 10.1128/AAC.02230-17 PMC6021653 29661871

[13] Halpern AB, Lyman GH, Walsh TJ, Kontoyiannis DP, Walter RB (2015). Primary antifungal prophylaxis during curative-intent therapy for acute myeloid leukemia. Blood.

[14] Suh HJ, Kim I, Cho JY, Park SI, Yoon SH, Hwang JH, Bae JY, Lee JO, Koh Y, Song KH (2018). Early therapeutic drug monitoring of Posaconazole Oral suspension in patients with hematologic malignancies. Ther Drug Monit.

[15] Bruggemann RJ, Alffenaar JW, Blijlevens NM, Billaud EM, Kosterink JG, Verweij PE, Burger DM (2009). Clinical relevance of the pharmacokinetic interactions of azole antifungal drugs with other coadministered agents. Clinical infectious diseases : an official publication of the Infectious Diseases Society of America.

[16] Scott LJ (2012). Micafungin: a review of its use in the prophylaxis and treatment of invasive Candida infections. Drugs.

[17] El-Cheikh J, Venton G, Crocchiolo R, Furst S, Faucher C, Granata A, Oudin C, Coso D, Bouabdallah R, Vey N (2013). Efficacy and safety of micafungin for prophylaxis of invasive fungal infections in patients undergoing haplo-identical hematopoietic SCT. Bone Marrow Transplant.

[18] Park HJ, Park M, Han M, Nam BH, Koh KN, Im HJ, Lee JW, Chung NG, Cho B, Kim HK (2014). Efficacy and safety of micafungin for the prophylaxis of invasive fungal infection during neutropenia in children and adolescents undergoing allogeneic hematopoietic SCT. Bone Marrow Transplant.

[19] van Burik JA, Ratanatharathorn V, Stepan DE, Miller CB, Lipton JH, Vesole DH, Bunin N, Wall DA, Hiemenz JW, Satoi Y (2004). Micafungin versus fluconazole for prophylaxis against invasive fungal infections during neutropenia in patients undergoing hematopoietic stem cell transplantation. Clinical infectious diseases : an official publication of the Infectious Diseases Society of America.

[20] Hirata Y, Yokote T, Kobayashi K, Nakayama S, Oka S, Miyoshi T, Akioka T, Hiraoka N, Iwaki K, Takayama A (2010). Antifungal prophylaxis with micafungin in neutropenic patients with hematological malignancies. Leukemia & lymphoma.

[21] Epstein DJ, Seo SK, Huang YT, Park JH, Klimek VM, Berman E, Tallman MS, Frattini MG, Papanicolaou GA (2018). Micafungin versus posaconazole prophylaxis in acute leukemia or myelodysplastic syndrome: a randomized study. The Journal of infection.

[22] Venton G, Adam H, Colle J, Labiad Y, Mercier C, Ivanov V, Suchon P, Fanciullino R, Farnault L, Costello R (2016). Micafungin as primary antifungal prophylaxis in patients presenting with acute myeloid leukemia. Medecine et maladies infectieuses.

[23] Suh HJ, Kim I, Cho JY, Park SI, Yoon SH, Lee JO, Koh Y, Song KH, Choe PG, Yu KS (2017). Comparison of plasma concentrations of Posaconazole with the Oral suspension and tablet in Korean patients with hematologic malignancies. Infection & chemotherapy.

[24] Shen Y, Huang XJ, Wang JX, Jin J, Hu JD, Yu K, Wu DP, Wang SJ, Yu L, Chen XQ (2013). Posaconazole vs. fluconazole as invasive fungal infection prophylaxis in China: a multicenter, randomized, open-label study. Int J Clin Pharmacol Ther.

[25] De Pauw B, Walsh TJ, Donnelly JP, Stevens DA, Edwards JE, Calandra T, Pappas PG, Maertens J, Lortholary O, Kauffman CA (2008). Revised definitions of invasive fungal disease from the European Organization for Research and Treatment of Cancer/invasive fungal infections cooperative group and the National Institute of Allergy and Infectious Diseases mycoses study group (EORTC/MSG) consensus group. Clinical infectious diseases : an official publication of the Infectious Diseases Society of America.

[26] Sohn HS, Lee TJ, Kim J, Kim D (2009). Cost-effectiveness analysis of micafungin versus fluconazole for prophylaxis of invasive fungal infections in patients undergoing hematopoietic stem cell transplantation in Korea. Clin Ther.

[27] Huang X, Chen H, Han M, Zou P, Wu D, Lai Y, Huang H, Chen X, Liu T, Zhu H (2012). Multicenter, randomized, open-label study comparing the efficacy and safety of micafungin versus itraconazole for prophylaxis of invasive fungal infections in patients undergoing hematopoietic stem cell transplant. Biology of blood and marrow transplantation : journal of the American Society for Blood and Marrow Transplantation.

[28] Lee CH, Lin JC, Ho CL, Sun M, Yen WT, Lin C (2017). Efficacy and safety of micafungin versus extensive azoles in the prevention and treatment of invasive fungal infections for neutropenia patients with hematological malignancies: a meta-analysis of randomized controlled trials. PLoS One.

[29] Inoue Y, Saito T, Ogawa K, Nishio Y, Kosugi S, Suzuki Y, Kato M, Sakai T, Takahashi M, Miura I (2012). Drug interactions between micafungin at high doses and cyclosporine a in febrile neutropenia patients after allogeneic hematopoietic stem cell transplantation. Int J Clin Pharmacol Ther.

[30] Moriyama B, Henning SA, Leung J, Falade-Nwulia O, Jarosinski P, Penzak SR, Walsh TJ (2012). Adverse interactions between antifungal azoles and vincristine: review and analysis of cases. Mycoses.

[31] Goto N, Hara T, Tsurumi H, Ogawa K, Kitagawa J, Kanemura N, Kasahara S, Yamada T, Shimizu M, Nakamura M (2010). Efficacy and safety of micafungin for treating febrile neutropenia in hematological malignancies. Am J Hematol.

[32] Leventakos K, Lewis RE, Kontoyiannis DP (2010). Fungal infections in leukemia patients: how do we prevent and treat them?. Clinical infectious diseases : an official publication of the Infectious Diseases Society of America.

[33] Pagano L, Caira M, Candoni A, Offidani M, Fianchi L, Martino B, Pastore D, Picardi M, Bonini A, Chierichini A (2006). The epidemiology of fungal infections in patients with hematologic malignancies: the SEIFEM-2004 study. Haematologica.

[34] Maertens J (2007). Evaluating prophylaxis of invasive fungal infections in patients with haematologic malignancies. Eur J Haematol.

[35] Bodey GP, Anaissie EJ, Elting LS, Estey E, O'Brien S, Kantarjian H (1994). Antifungal prophylaxis during remission induction therapy for acute leukemia fluconazole versus intravenous amphotericin B. Cancer.

[36] NCCN Clinical Practice Guidelines in Oncology (NCCN Guidelines®), Acute Lymphoblastic Leukemia, Version 1.2018, NCCN.org, 3/12/2018. [https://www.nccn.org/professionals/physician_gls/pdf/all.pdf].

